# Selection of Reference Genes for Quantitative Real-Time RT-PCR Studies in Tomato Fruit of the Genotype MT-*Rg1*

**DOI:** 10.3389/fpls.2016.01386

**Published:** 2016-09-13

**Authors:** Karla L. González-Aguilera, Carolina F. Saad, Ricardo A. Chávez Montes, Marcio Alves-Ferreira, Stefan de Folter

**Affiliations:** ^1^Unidad de Genómica Avanzada – Laboratorio Nacional de Genómica para la Biodiversidad, Centro de Investigación y de Estudios Avanzados del Instituto Politécnico NacionalIrapuato, Mexico; ^2^Laboratório de Genética Molecular Vegetal, Universidade Federal do Rio de JaneiroRio de Janeiro, Brazil

**Keywords:** qRT-PCR, expression analysis, reference genes, tomato (*Solanum lycopersicum*), fruit development

## Abstract

Quantitative real-time RT-PCR (qRT-PCR) has become one of the most widely used methods for accurate quantification of gene expression. Since there are no universal reference genes for normalization, the optimal strategy to normalize raw qRT-PCR data is to perform an initial comparison of a set of independent reference genes to assess the most stable ones in each biological model. Normalization of a qRT-PCR experiment helps to ensure that the results are both statistically significant and biologically meaningful. Tomato is the model of choice to study fleshy fruit development. The miniature tomato (*Solanum lycopersicum* L.) cultivar Micro-Tom (MT) is considered a model system for tomato genetics and functional genomics. A new genotype, containing the *Rg1* allele, improves tomato *in vitro* regeneration. In this work, we evaluated the expression stability of four tomato reference genes, namely *CAC*, *SAND*, *Expressed*, and *ACTIN2.* We showed that the genes *CAC* and *Exp* are the best reference genes of the four we tested during fruit development in the MT-*Rg1* genotype. Furthermore, we validated the reference genes by showing that the expression profiles of the transcription factors *FRUITFULL1* and *APETALA2c* during fruit development are comparable to previous reports using other tomato cultivars.

## Introduction

Reverse transcription followed by quantitative PCR (qRT-PCR) assay is an extremely sensitive technique that provides accurate and reproducible quantification of nucleic acids based on the exponential incorporation of fluorescent molecules into genetic material (Gachon et al., 2004; Nolan et al., 2006). Nowadays, qRT-PCR analysis has become the method of choice for gene expression studies and validating transcriptomic data. One of the most crucial points in RT-qPCR data analysis is the choice of a proper normalization method. The purpose of normalization is to correct for variability associated with the experimental procedure, such as the amount of starting material, RNA extraction and enzymatic efficiencies, and differences in overall transcriptional activity between tissues or cells (Expósito-Rodríguez et al., 2008; Gutierrez et al., 2008a,b; Udvardi et al., 2008). To date, the parallel quantification of endogenous reference genes is accepted as the most reliable method for sample normalization. The normalization of relative quantities with reference genes relies on the assumption that the reference genes are stably expressed across all tested samples, and that they are not being significantly altered across treatments or conditions (Vandesompele et al., 2002). However, several reports have shown that the transcript levels of commonly used reference genes, known as housekeeping genes, can vary considerably under different experimental conditions (e.g., Thellin et al., 1999; Suzuki et al., 2000; Czechowski et al., 2005; Jain et al., 2006). Moreover, a reference gene with stable expression in one organism may not be suitable for normalization of gene expression in another organism (e.g., Jain et al., 2006; Jian et al., 2008; Manoli et al., 2012; Lambret-Frotté et al., 2015; Kanakachari et al., 2016).

Since there are no universal reference genes, the optimal strategy to normalize raw qRT-PCR data is to perform an initial comparison of a set of independent reference genes to assess the most stable ones in each particular experimental background or biological model. The use of multiple reference genes does not only produce more reliable data, but permits an evaluation of the stability of the reference genes themselves. In this way, normalization of a qPCR assay helps to ensure that the results are both statistically significant and biologically meaningful (Hellemans et al., 2007; Expósito-Rodríguez et al., 2008; Gutierrez et al., 2008a,b; De Spiegelaere et al., 2015).

Fruits are an important evolutionary acquisition of angiosperms, which convey protection to seeds and ensure their optimal dispersal in the environment. Tomato is the model of choice to study fleshy fruit development (Seymour et al., 2013; Pesaresi et al., 2014). The miniature tomato or micro tomato (*Solanum lycopersicum* L.) cultivar Micro-Tom (MT) is considered a model system for tomato genetics and functional genomics (Scott and Harbaugh, 1989; Meissner et al., 1997; Emmanuel and Levy, 2002; Dan et al., 2006; Sun et al., 2006; Carvalho et al., 2011). This variety displays convenient traits for plant research, such as short life cycle, miniature size, high density growth, and the suitability for transgenic plant production and large scale mutagenesis (Meissner et al., 1997; Emmanuel and Levy, 2002; Watanabe et al., 2007; Carvalho et al., 2011).

In order to enhance tomato genetic transformation, Pino et al. (2010) developed a MT near-isogenic genotype harboring the *Rg1* allele from *S. peruvianum*. This new genotype, MT-*Rg1*, allows reduced exposure to exogenous hormone applications during transformation protocols, improving *in vitro* regeneration (Pino et al., 2010).

Although tomato has become an important model for genetic and molecular studies in fleshy fruits, there are few examples in literature where best reference genes have been identified and validated in tomato. The best example is by Expósito-Rodríguez et al. (2008), with a focus on different tomato tissues. A few other studies report on appropriate reference gene selection for expression analysis in tomato seeds (Dekkers et al., 2012) and pathogen infected tomato plants (Lacerda et al., 2015; Muller et al., 2015). Therefore, there is still a need for more studies on appropriate reference gene selection for expression studies during different conditions and developmental processes, for instance tomato fruit development.

In this work, we evaluated the expression stability of four commonly used tomato reference genes, namely *CAC*, *SAND*, *Expressed*, and *ACTIN2*, and evaluated the expression of the transcription factors *FRUITFULL1* (*FUL1*) and *APETALA2c* (*AP2c*) across eight MT-*Rg1* fruit developmental stages. Our results allowed us to select suitable reference genes for qRT-PCR studies during fruit development, and show that the expression profiles of *FUL1* and *AP2c* are similar to those previously reported for other tomato fruit cultivars.

## Results

### Biological Samples and Candidate Reference Genes

**FIGURE 1:**
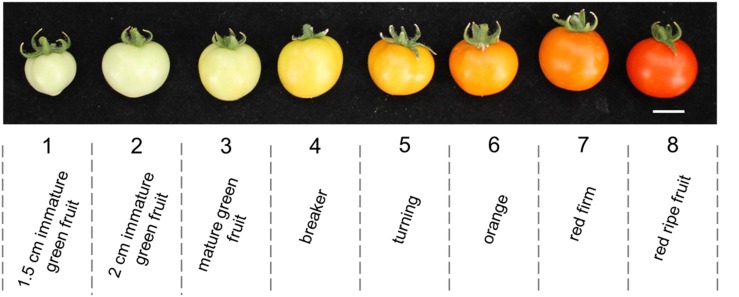
***MT-Rg1* fruit developmental stages used for gene expression analysis in this study.** Scale bar: 1 cm.

Tomato Micro-Tom *Rg1* (MT-*Rg1*) fruits were sampled at eight different developmental stages (**Figure 1**), according to Gillaspy et al. (1993). The collected fruit stages are as follows: (1) 1.5 cm immature green fruit, (2) 2 cm immature green fruit, (3) mature green fruit, (4) breaker, (5) turning, (6) orange, (7) red firm, and (8) red ripe fruit. Fruits (pericarp tissue only) were frozen and stored at –80°C until further use. Total RNA was isolated using Trizol, followed by several precipitation steps, a DNase I treatment, and subsequently, cDNA was prepared, as described in the Materials and Methods section.

Based on previous studies conducted on *S. lycopersicum* cv. ciliegia (Expósito-Rodríguez et al., 2008), a total of four candidate reference genes were selected for qRT-PCR normalization. These are *CAC*, *SAND*, and *Expressed* (Expósito-Rodríguez et al., 2008), together with the commonly used house-keeping gene *ACTIN2* (Bemer et al., 2012) (**Table 1**). Primers used are listed in **Table 2**. The primers for the first three genes span intronic regions (**Table 2**), i.e., a larger amplicon will be seen when DNA contamination is present. Primer melting curves for all genes showed a unique peak corresponding to the expected amplicon (Supplementary Figure 1). The correct size of the amplicons was verified by gel electrophoreses.

**Table 1:** Candidate reference genes and validation genes used for qPCR expression study in *Solanum lycopersicum* cv. MT-*Rg1* during fruit development stages.

Symbol	Gene name	Gene ID	Locus description/Function
*CAC*	Clathrin adaptor complexes medium subunit	Solyc08g006960	Intracellular trafficking. Endocytic pathway.
*SAND*	*SAND* family	Solyc03g115810	Endocytosis. Ion transport and homeostasis.
*Expressed*	Expressed sequence	Solyc07g025390	Gene expression
*ACT2*	*ACTIN2*	Solyc11g005330	Cytoskeletal protein
*AP2c*	*APETALA2c*	Solyc02g093150	AP2-like ethylene responsive transcription factor. Fruit ripening.
*FUL1*	*FRUITFULL1*	Solyc06g069430	MADS-box transcription factor. Flower and fruit development.


**Table 2:** Details of primers of candidate reference genes, validation genes and parameters derivated from qPCR analysis.

Gene	Sequence (5′–3′)	Transcript ID^∗^	Amplicon length (bp)		*T*m (°C)	Efficiency	
					
			cDNA	genomic		Mean^∗∗^	*SD*
*CAC*	CCTCCGTTGTGATGTAACTGG	Solyc08g006960.2.1	173	592	55.5	0.879375	0.0118106
	ATTGGTGGAAAGTAACATCATCG				53.5		
*SAND*	TTGCTTGGAGGAACAGACG	Solyc03g115810.2.1	164	3559	55.1	0.906342	0.019498
	GCAAACAGAACCCCTGAATC				54.1		
*Expressed*	GCTAAGAACGCTGGACCTAATG	Solyc07g025390.2.1	183	291	55.6	0.878654	0.011885
	TGGGTGTGCCTTTCTGAATG				55.6		
*ACT2*	CATTGTGCTCAGTGGTGGTTC	Solyc11g005330.1.1	176	176	56.5	0.864061	0.0238442
	TCTGCTGGAAGGTGCTAAGTG				57.0		
*AP2c*	CCGTTTCGAATTCAAGTTCA	Solyc02g093150.2.1	122	122	51.1	0.874625	0.00794325
	ACCCAGACCCACCATAGAGA				57.2		
*FUL1*	GTTTTGCCACAACAACTGGACTC	Solyc06g069430.2.1	106	1124	57.0	0.83762	0.0191408
	CTTGCTGCTGTGAAGAACTACC				56.0		


*^∗^Transcript ID from Sol Genomics Network (SL2.50 ITAG2.4). ^∗∗^Mean of the two biological replicates.*

Finally, as described at the end of the Results section, relative expression of the transcription factors *FUL1* (Bemer et al., 2012) and *AP2c* (Karlova et al., 2011) was analyzed and compared to previous reports (Karlova et al., 2011; Bemer et al., 2012) in order to validate the normalization procedure using the most stable reference genes identified during fruit development in MT-*Rg1*.

### Expression Stability of Candidate Reference Genes

**FIGURE 2:**
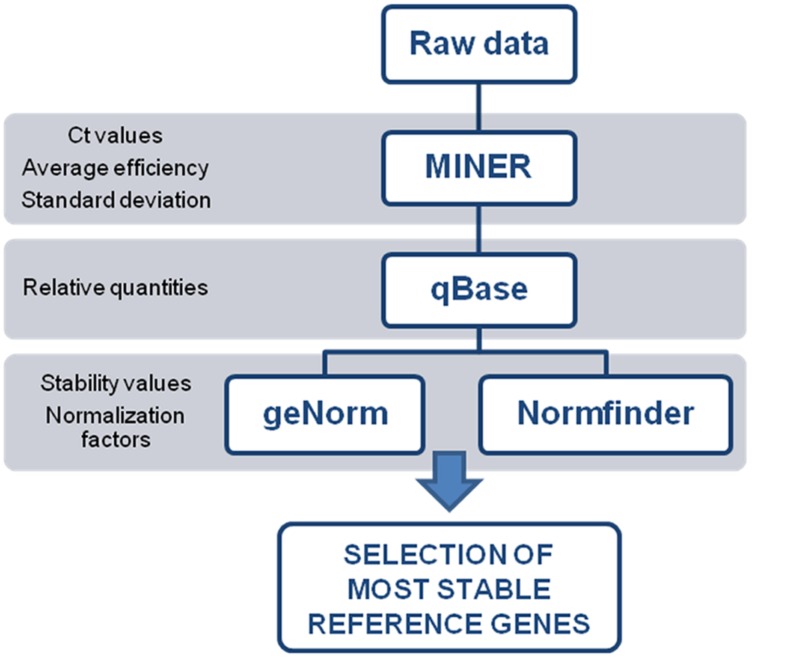
**Data processing workflow for quantitative PCR data.** Each program generates an input file needed for next step in the workflow.

Data processing is illustrated in **Figure 2**. To evaluate the individual reaction kinetics, without the need for a standard curve, we used Real-Time PCR Miner software (Zhao and Fernald, 2005). Raw data of the time (cycle) sequence fluorescence values were imported from the real time PCR machine into Miner to calculate primer efficiency and its associated standard deviation, and the cycle threshold (Ct) value. The resulting data was used as input for qBase.

The qBase software (Hellemans et al., 2007) processes data modules as independent experiments. Thus Ct values for each individual reaction, and each tested reference gene, were imported as independent experiments. The first step in the qBase workflow combines raw data from all individual run files of the same experiment into a single data table, where data points with identical sample and gene names are automatically identified as technical replicates. Then, we executed a data quality control, where replicated reactions that differ between them in more than 0.5 Ct were excluded from further analysis as a potential outlier, totaling 19 excluded cases (6.59% of the complete data set). Next, average efficiency of genes and its associated standard deviation were replaced with the values obtained from the Miner software for each gene. Finally, we performed the quantification of relative quantities, which lie in the conversion of quantification cycle values into relative quantities based on the gene specific amplification efficiency.

Then a normalization factor based on the expression levels of the tested reference genes was calculated using both geNorm (Vandesompele et al., 2002) and NormFinder (Andersen et al., 2004), which are Excel based software packages.

**FIGURE 3:**
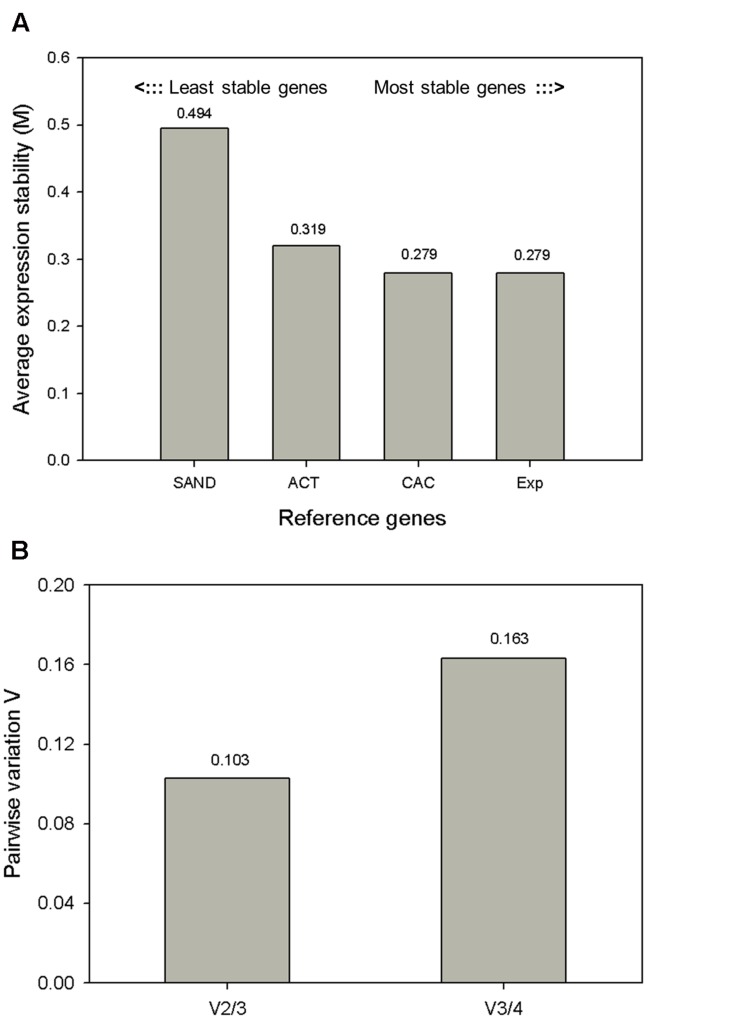
**Expression stability values (M) and pairwise variation analysis of candidate reference genes calculated by geNorm.** **(A)** geNorm average expression stability (*M*) of the four candidate reference genes. Lower values indicate increased gene stability across samples. In total 16 samples (including both biological replicates) were included in this analysis. **(B)** The geNorm pairwise variation (*V*) was analyzed between the normalization factors (NF). Using two reference genes instead of three genes (V2/3) resulted in a value below the cutoff of 0.15, i.e., that the use of two reference gene is sufficient for normalization.

geNorm calculates a gene stability measure *M* as the average pairwise variation of a particular gene against other reference genes (Vandesompele et al., 2002) (**Figure 3A**). Genes are ranked according to increasing expression stability, with *CAC* and *Exp* displaying the most stable expression profiles and *ACT* and *SAND* less stable profiles. The *CAC* and *Exp* genes were also reported as one of the most stable reference genes in *S. lycopersicum* cv. ciliegia (Expósito-Rodríguez et al., 2008). Next, normalization factors were calculated for the most stable reference genes. Additionally, the software calculates the pairwise variation (*V*) between two sequential normalization factors (NF*_n_* and NF_*n* + 1_) in order to determine the minimum number of reference genes required for normalization (**Figure 3B**) (Vandesompele et al., 2002). In other words, pairwise variation estimates how the NF changes if you add another reference gene to the NF calculation. geNorm pairwise comparison shows that adding a third gene has no significant contribution to the newly calculated normalization factor (V2/3), meaning that using two reference genes is sufficient to normalize our qRT-PCR data. Its value is lower than the cutoff of 0.15 proposed by Vandesompele et al. (2002), which suggests the inclusion of additional reference genes are not necessary. From this analysis we concluded the use of the two most stable reference genes *CAC* and *Exp* is sufficient for accurate normalization (*V*2/3 = 0.103) in MT-*Rg1* fruits.

**FIGURE 4:**
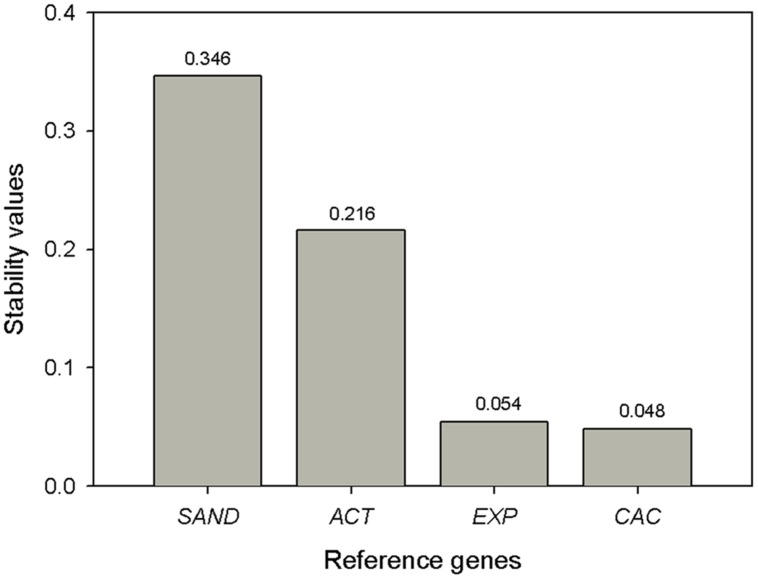
**NormFinder expression stability values (*M*) and ranking of the candidate reference genes.** Lower values indicate more stable expression.

NormFinder (Andersen et al., 2004) is an algorithm to identify the optimal normalization gene among a set of candidates. The validity of this approach is related to the number of samples and candidates analyzed because it considers intra- and intergroup variation to estimate a stability value of the tested reference genes. The intragroup refers to the biological replicates and the intergroup refers to the different fruit developmental stages. We used the input file containing the qBase-calculated relative quantity values for each gene, sample and biological replica and ran the analysis. The results revealed *CAC* as the most stable reference gene with the best stability value (i.e., the lowest value) of 0.049 (**Figure 4**). The best combination of genes was *CAC* and *Exp*. These results match those proposed by the geNorm software.

Consequently, we decided to continue the experiment using the best reference genes *CAC* and *Exp* to normalize the gene expression data of *FUL1* and *AP2c* and compare it with normalized expression data with the less stable reference gene *SAND* in all tested samples.

### *AP2c* and *FUL1* Expression Profiles

To assess the validity of the MT-*Rg1* genotype as a model tomato species, we calculated the relative expression of *AP2c* and *FUL1* at different developmental stages, and compared them with previous reports (Karlova et al., 2011; Bemer et al., 2012). Accurate measurement of gene expression requires a normalization by multiple, carefully selected reference genes. We therefore decided to normalize gene expression using both the most stable reference gene pair identified by GeNorm and NormFinder, *CAC* and *Exp*, but also using the less stable reference gene, *SAND*.

**FIGURE 5:**
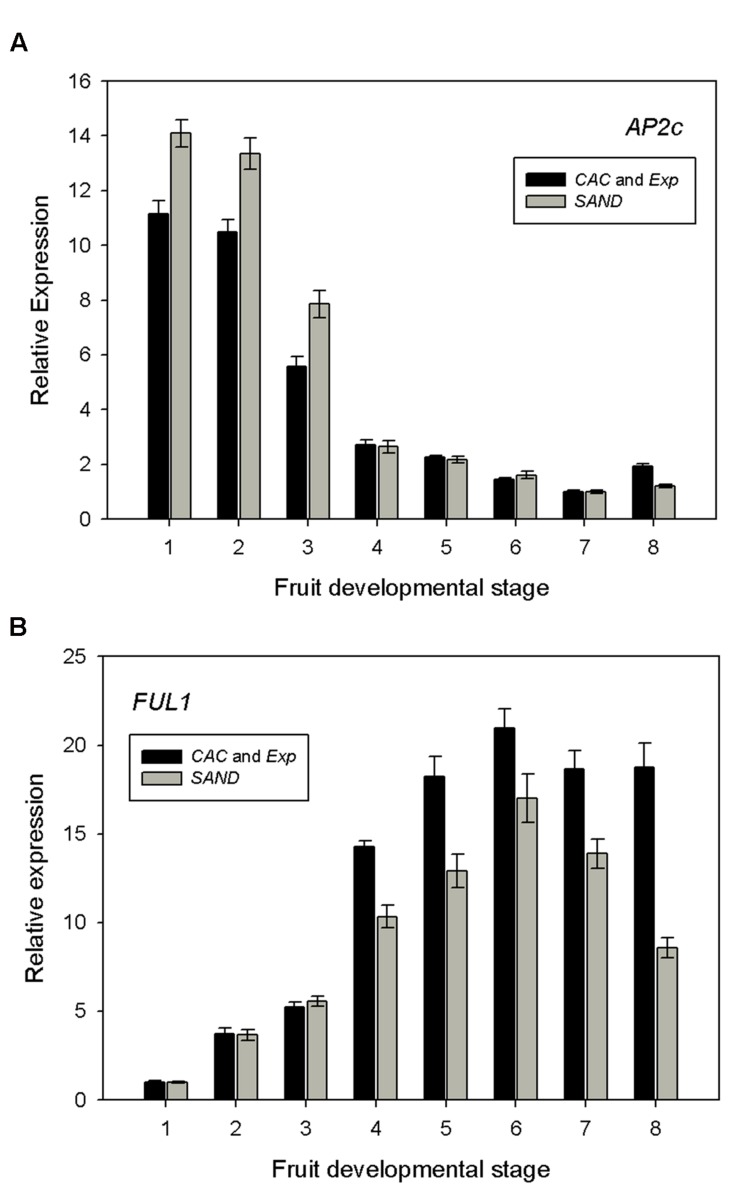
**Expression profiles of **(A)***AP2c* and **(B)***FUL1* in *MT-Rg1* fruits.** Dataset was normalized using the two best reference genes (*CAC* and *Exp*) and compared with normalized data using the poorly ranked reference gene (*SAND*). Error bars represent the standard error of the mean.

*AP2* is a transcription factor which plays important roles in development, ethylene response and pathogen resistance (Karlova et al., 2011). Five distinct tomato cDNAs encoding *AP2* putative tomato orthologs were identified by Karlova et al. (2011) in the Moneymaker tomato cultivar. From this report, we chose the *AP2c* sequence, which shows the highest expression in early stage fruits, but no significant expression beyond the mature green stage. Our results in MT-*Rg1* fruits (**Figure 5A**) were concordant with the previously reported expression profile in the tomato cultivar Moneymaker (Karlova et al., 2011). In the first three fruit stages, we observed a high expression and then expression levels dropped from the breaker stage on.

Tomato has two orthologs of *FUL*, a transcription factor involved in fruit ripening (Bemer et al., 2012). We also calculated the relative expression of *FUL1* in order to validate our MT-*Rg1* qRT-PCR results. *FUL1* expression was found to be very low during early stages of fruit development, but rapidly increased from the breaker stage, reaching its maximum in the orange ripe stage (**Figure 5B**). A comparable trend of expression was reported in tomato MT fruits (Bemer et al., 2012). Normalizing the qRT-PCR results for *AP2c* and *FUL1* with the less stable reference gene *SAND* did result in differences in expression levels in the fruit stages with high *AP2c* and *FUL1* expression levels, although the trend of the expression profile did not change substantively.

## Discussion

Several reports have shown the importance of selecting proper reference genes for data normalization, and how the identity of these genes will vary depending on the model of study (Jain et al., 2006; Expósito-Rodríguez et al., 2008; Jian et al., 2008; Manoli et al., 2012; Lambret-Frotté et al., 2015; Kanakachari et al., 2016). Although reference genes have been identified for the *S. lycopersicum* cv. ciliegia, there are no reports about the most reliable reference genes for the MT-*Rg1* tomato genotype.

This work constitutes an effort to validate appropriate reference genes for the quantification of transcript levels by quantitative RT-PCR in the MT-*Rg1* tomato genotype. We have tested the expression stabilities of four reference candidate genes (*CAC*, *SAND*, *Expressed*, and *ACT2*) in a set of eight developmental tomato MT-*Rg1* fruit stages.

Correct sample normalization is an absolute prerequisite for reliable and accurate measurement of gene expression. The optimal strategy to normalize raw qPCR data is to perform an initial comparison of a set of independently regulated reference genes to assess the most stable ones in each specific experiment or biological setting (e.g., Czechowski et al., 2005; Expósito-Rodríguez et al., 2008; Gutierrez et al., 2008a; De Spiegelaere et al., 2015).

Because of the increasing attention on a proper normalization of qRT-PCR data, there are increasing number of methods and software packages that have been developed for the validation of the most stable reference genes. Specialized software like geNorm (Vandesompele et al., 2002) and NormFinder (Andersen et al., 2004) are excellent tools to determine best reference genes when a new system or tissue is used. Reference genes should be established for each tested tissue, allowing a better interpretation and biological significance. Unstable reference genes, if used for normalization, can radically change the expression pattern of a given gene under study causing errors in results and, thereby, the interpretation or understanding of gene function (Gutierrez et al., 2008a,b). This emphasizes the importance of preliminary evaluation studies, aimed to identify the most stable reference genes in different organism and also between different tissues of the same species.

The suitability of reference genes identified in this study (*CAC* and *Exp*) was validated through an assessment of the expression profiles of two transcription factors. The expression of *AP2c* and *FUL1* during fruit development in the MT-*Rg1* genotype showed an activity similar to other tomato cultivars (Karlova et al., 2011; Bemer et al., 2012).

In summary, here we showed that the genes *CAC* and *Exp* are appropriate reference genes during fruit development in the MT-*Rg1* genotype. Furthermore, we validated the normalization method for the tomato fruit developmental genes *AP2c* and *FUL1*.

## Materials and Methods

### Plant Growth Conditions

Tomato (*S. lycopersicum*) MT-*Rg1* genotype (Pino et al., 2010) seeds were sown in flat trays containing a 3:1:1:1 mixture of leaf soil, soil, sand, and perlite, respectively. Plants were grown in a greenhouse under local conditions (20°43′1″ N; 101°19′56″) in the summer (around 16 h light/8 h dark) at an average mean temperature of 30°C.

### Tissue Collection

Tomato fruits were sampled at eight different developmental stages, based on the fruit stage division proposed by Gillaspy et al. (1993). In total, four fruits of each developmental stage were collected: 1.5 cm immature green fruits, 2 cm immature green fruit, mature green fruit, breaker, turning, orange, red firm, and red ripe (**Figure 1**). Fruits devoid of seeds and placental tissue (only pericarp tissue) were frozen in liquid nitrogen and stored at -80°C until RNA extraction.

### RNA Extraction and cDNA Synthesis

Total RNA extraction was performed using the TRIzol^®^ (Ambion) protocol, followed by precipitation using 0.8 M sodium citrate, 1.2 M NaCl and isoproponol, a second precipitation using 8 M LiCl, and a third precipitation with 3 M NaAc pH 5.2 and 100% ethanol. Subsequently, a DNase I (Life Technologies) treatment was performed according to manufacturer’s specifications, and RNA was recovered using phenol/chloroform extraction followed by a precipitation with 3 M NaAc pH 5.2 and 100% ethanol. RNA was resuspended in 10 μl of DEPC water and quantified by measuring its absorbance at 260 nm. RNA integrity was evaluated by the 260/280 and 260/230 ratios, and confirmed by agarose gel electrophoresis.

cDNA was synthesized from 1.3 μg of total RNA using the SuperScript^®^ III System (Life Technologies). In summary, total RNA was mixed with 1 μl of oligo dT (50 μM), 1 μl of dNTPs (10 μM) and MQ water, giving a total volume of 14 μl, and incubated for 5 min at 65°C and then chilled on ice. Subsequently, 4 μl of First Strand Buffer (5x), 1 μl of DTT (0.1 M) and 1 μl of SuperScript reverse transcriptase III (200 units/ μl) were added, each reaction was incubated for 2 h at 50°C and, finally, inactivated for 5 min at 70°C. cDNA was diluted 1:50 for use in quantitative real-time PCR experiments.

### Quantitative Real-Time PCR

Quantitative real-time PCR amplification reactions were performed and run in technical triplicate on 96-wells plates on an ABI 7500 Fast Real Time PCR System using SYBR^®^ Green I (Life Technologies). Each PCR reaction mix consisted of 2 μl of SYBR Green (1:10000), 0.4 μl of forward and reverse oligos (10 mM), 2 μl of PCR buffer (10x), 0.05 μl of dNTPs (10 mM), 1.2 μl of MgCl_2_ (50 mM) and 0.05 μl of Platinum^®^ Taq DNA Polymerase (Life Technologies; 2 U/rxn) in a total volume of 10 μl. Finally, 10 μl of 1:50 diluted template cDNA was added, resulting in a total volume of 20 μl per PCR reaction. PCR cycling was performed as follows: 5 min at 94°C followed by 40 rounds of 15 s at 94°C, 10 s at 60°C, 15 s at 72°C, and finally 1 round of 35 s at 60°C. Melting curve cycling consisted of: 15 s at 95°C, 1 min at 60°C, 30 s at 95°C, and 15 s at 60°C.

The qRT-PCR experiment was performed using two biological replicates. For the first one, total RNA extracted from one fruit of each stage was used. cDNA for the second biological replicate was prepared from total RNA extracted from a pool of three fruits of each stage. For each analysis, three technical replicates were performed. The Supplementary Figure 2 shows the results of the comparison between the two biological replicates and the standard deviation of the three technical replicates for each fruit stage. In general, the detected Ct values did not differ much, but slightly more stability was observed in those where the cDNA was prepared from the RNA of the three pooled fruits. No difference in the standard deviation was observed between the two strategies (Supplementary Figure 2).

### Data Analysis

Raw fluorescence values from quantitative PCR experiments were imported into the Real-Time PCR Miner software (Zhao and Fernald, 2005). Ct values, average efficiency and standard deviation calculated by the Miner program were used as input for the qBase software (Hellemans et al., 2007). The qBase program calculates relative quantities of expression and produced an input file for the geNorm software (Vandesompele et al., 2002). The geNorm program determines the minimum number of genes required to calculate a reliable normalization factor. Finally, the qBase data file, with few modifications, was also used as an input file for the NormFinder program (Andersen et al., 2004), which considers the average expression stability between replicates and also between samples (intra- and inter-group variation, respectively) to suggest the best candidates for reference genes.

## Author Contributions

KG-A and CS performed experimental work and data analysis. KG-A, CS, MA-F, and SdF conceived the project and designed the experiments. KG-A and RACM made the figures. KG-A, RACM, and SdF drafted the manuscript. All authors read and approved the final manuscript version.

## Conflict of Interest Statement

The authors declare that the research was conducted in the absence of any commercial or financial relationships that could be construed as a potential conflict of interest.
