# Supplementary material for: Selection of Reference Genes for Quantitative Real-Time RT-PCR Studies in Tomato Fruit of the Genotype MT-Rg1
Source: Front Plant Sci. 2016 Sep 13;7:1386. doi: 10.3389/fpls.2016.01386 (PMC5021083; doi:10.3389/fpls.2016.01386)
Supplement: Supplementary file 1 [file Data_Sheet_1.PDF]

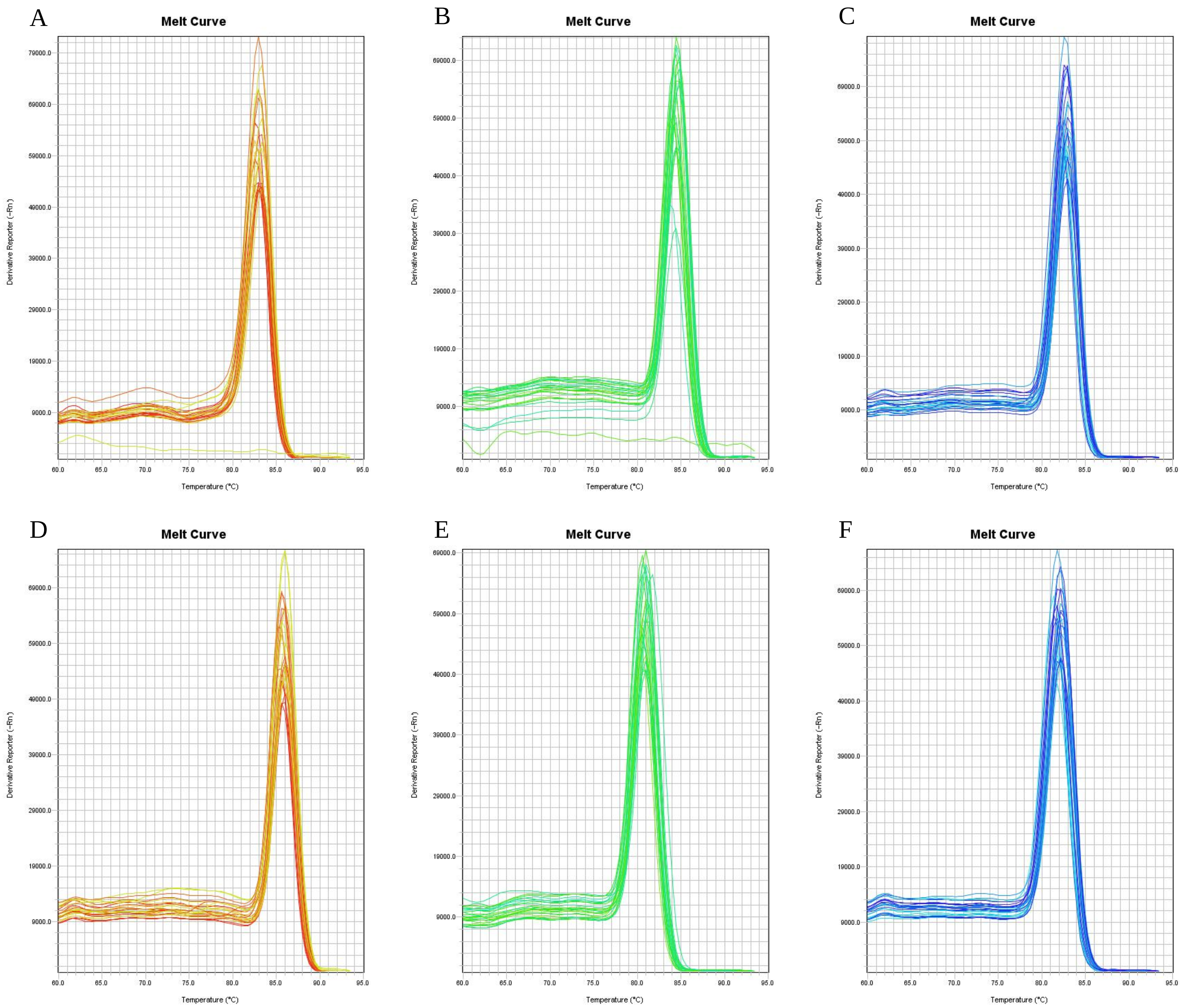

Supplementary Figure 1. Melting curves of analyzed genes: *CAC* (A), *SAND* (B), *Expressed* (C), *ACT2* (D), *AP2c* (E), and *FUL1* (F). All primer pairs resulted in a unique peak corresponding to only one amplicon.
