# Supplementary material for: Selection of Reference Genes for Quantitative Real-Time RT-PCR Studies in Tomato Fruit of the Genotype MT-Rg1
Source: Front Plant Sci. 2016 Sep 13;7:1386. doi: 10.3389/fpls.2016.01386 (PMC5021083; doi:10.3389/fpls.2016.01386)
Supplement: Supplementary file 2 [file Data_Sheet_2.PDF]

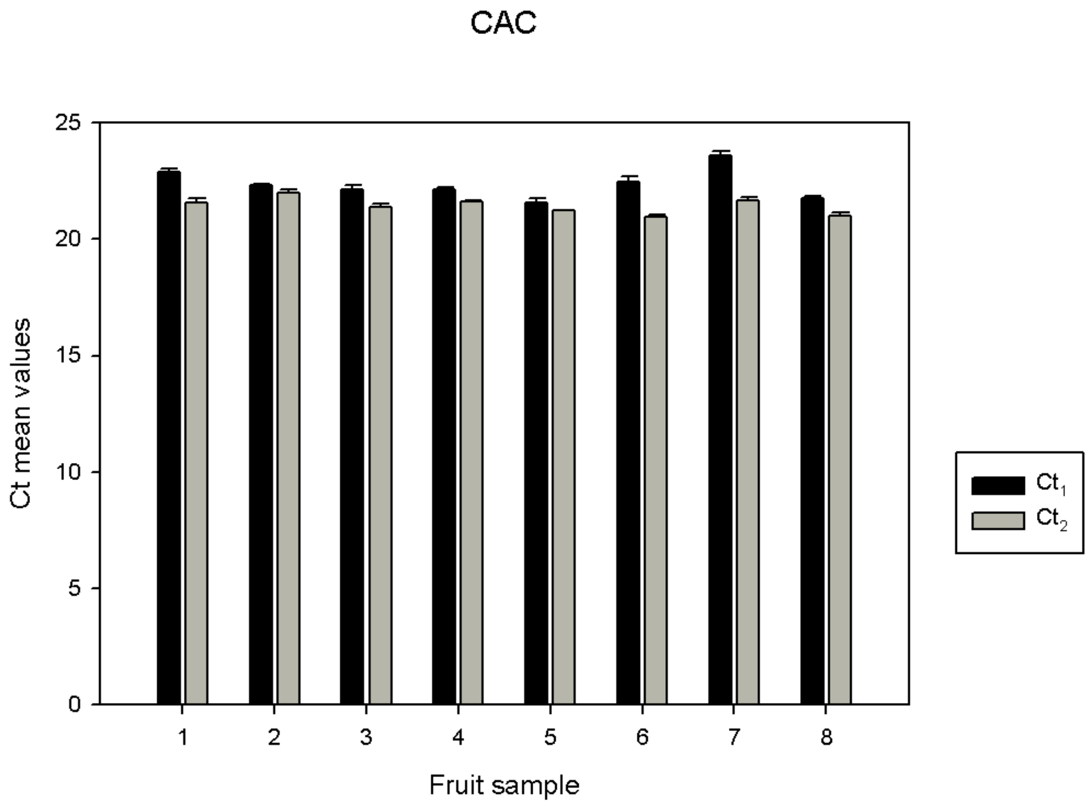

CAC
25
20
15
10
5
0
Ct mean values
1
2
3
4
5
6
7
8
Fruit sample
Ct1
Ct2

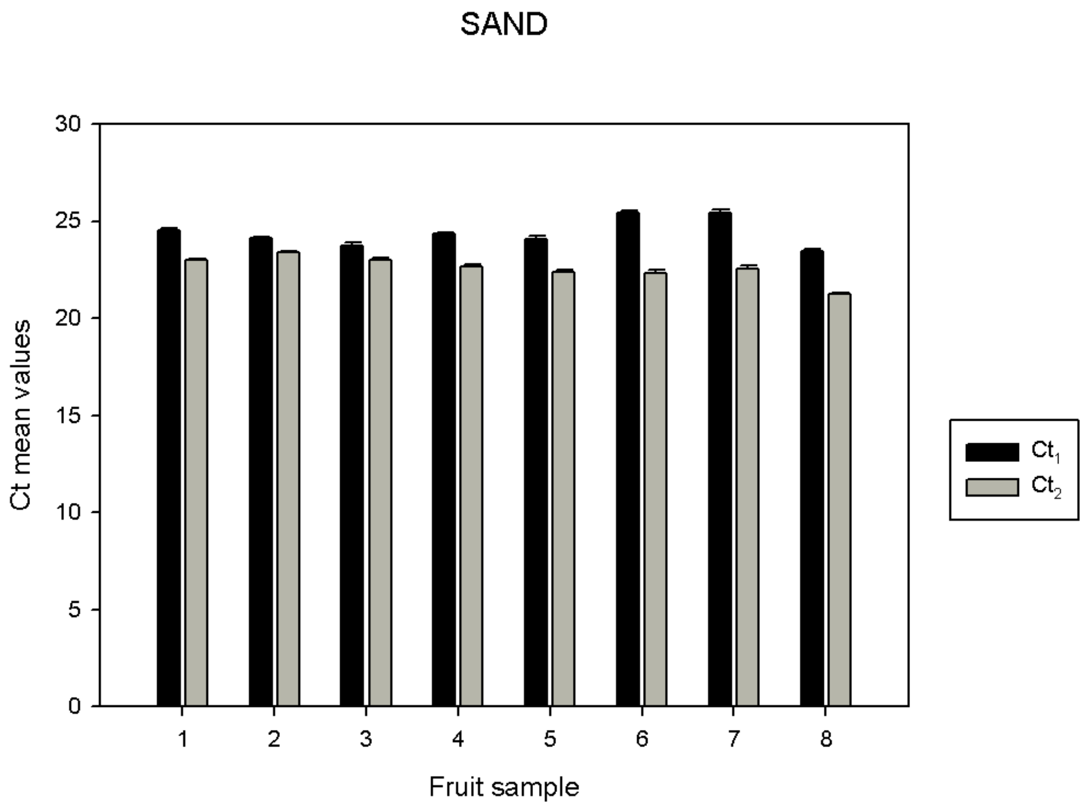

SAND
30
25
20
15
10
5
0
Ct mean values
1
2
3
4
5
6
7
8
Fruit sample
Ct1
Ct2

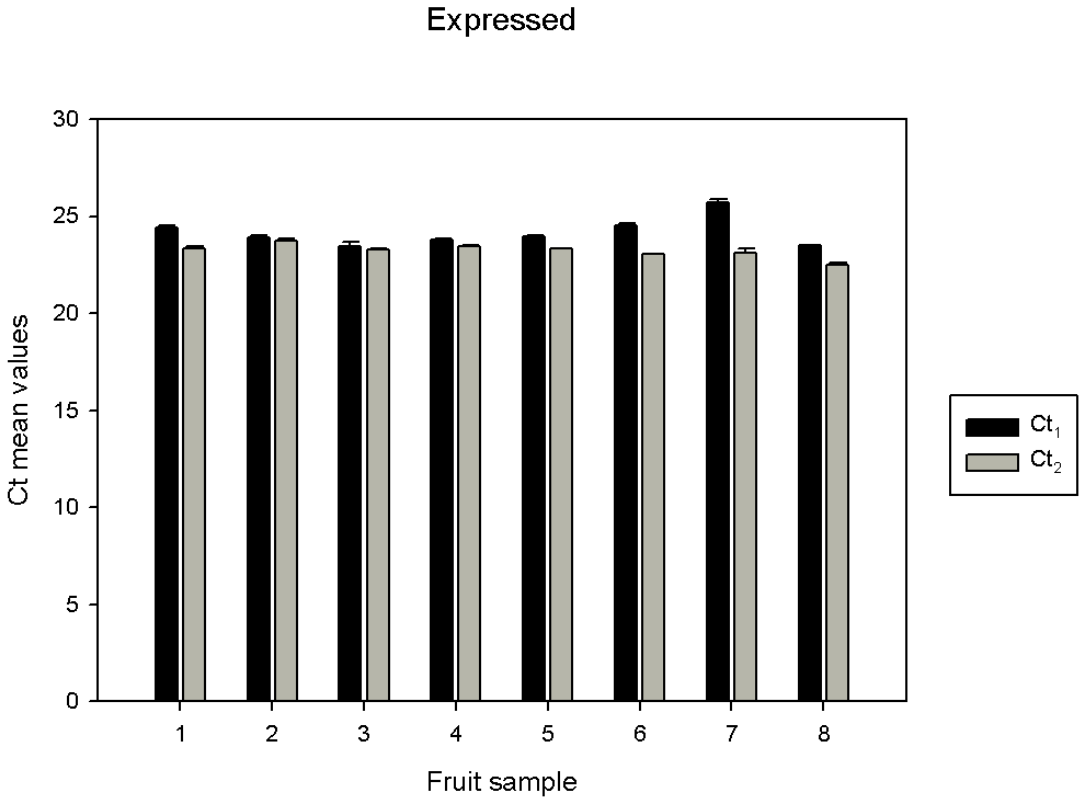

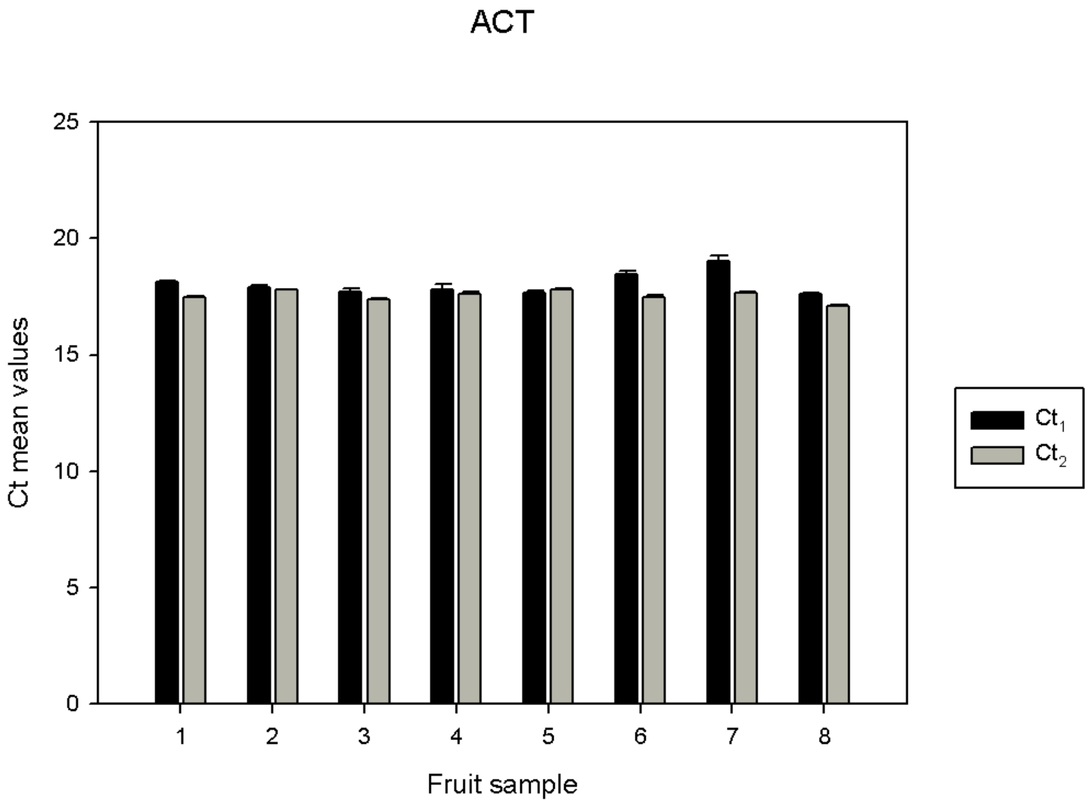

Supplementary Figure 2. Comparison of Ct values between the two biological replicates (Ct_1: single fruit; Ct_2: three pooled fruits) for the reference genes: *CAC*, *SAND*, *Expressed*, *ACT2*. Numbers 1 to 8 indicate fruit stage. Error bars represent the standard deviation of the technical replicates.
